# Effects of different nitrogen levels on growth and rhizosphere soil microorganisms of *Idesia polycarpa* Maxim

**DOI:** 10.3389/fpls.2026.1743337

**Published:** 2026-02-19

**Authors:** Chao Miao, Yigeng Zhu, Xiaoyu Wu, Yi Yang, Qiupeng Yuan, Zuwei Hu, Wenwen Zhong, Chen Chen, Tao Zhang, Zhen Liu, Yanmei Wang, Xiaodong Geng, Qifei Cai, Li Dai, Juan Wang, Yongyu Ren, Fangming Liu, Xianlong Rao, Hanjian Hu, Tailin Zhong, Zhi Li

**Affiliations:** 1 College of Forestry, Henan Agricultural University, Zhengzhou, China; 2 College of Urban Construction, Zhejiang Shuren University, Shaoxing, China; 3 National Forestry and Grassland Administration Key Laboratory for Central Plains Forest Resources Cultivation, Zhengzhou, China; 4 Henan Province Engineering Technology Research Center for Idesia, Zhengzhou, China; 5 Henan Vocational College of Agriculture, Zhengzhou, China; 6 Subtropical Forestry Experimental Center, Chinese Academy of Forestry, Fenyi, China

**Keywords:** metabolic pathways, rhizosphere, root system, soil microorganism, soil physicochemical properties

## Abstract

**Aims:**

*Idesia polycarpa* Maxim is a valuable oil and timber species, yet scientific guidance for its fertilization management remains scarce, limiting its productivity. This study aims to investigate the effects of different nitrogen (N) fertilizer levels on the growth characteristics of *I. polycarpa* to provide a theoretical basis for its fertilization management.

**Methods:**

Uniformly healthy one-year-old seedlings of *I. polycarpa* were treated with four nitrogen (N) application rates: 0 (control, CK), 1.2 (low nitrogen, LN), 2.4 (medium nitrogen, MN), and 3.6 (high nitrogen, HN) grams per plant. To assess the rhizosphere microbial community, high-throughput sequencing was performed targeting the bacterial 16S rRNA and fungal ITS gene regions.

**Results:**

The results demonstrated that N fertilization significantly enhanced plant growth and soil physicochemical properties compared to the CK treatment. Specifically, the MN treatment significantly increased root length, root volume, and root surface area (*p* < 0.05). The average root diameter was also higher in all N-fertilized groups than in CK. N application influenced soil properties: the HN treatment resulted in lower soil pH but higher alkali-hydrolyzable nitrogen (AN) and available potassium (AK) content, while the MN treatment exhibited higher soil organic matter (SOM) and available phosphorus (AP) content. The soil bacteria community was dominated by Proteobacteria, Chloroflexota, Acidobacteria, and Actinobacteria, While Ascomycota dominates the fungal community.

**Conclusion:**

The study found that the primary metabolic pathway of bacteria in the rhizosphere soil of *I. polycarpa* was metabolism, while the main metabolic pathways of fungi were biosynthesis, precursor metabolism, and energy synthesis. Furthermore, an N application rate of 1.2–2.4 g per plant per month is recommended for optimal growth during the early rapid growth phase of *I. polycarpa*.

## 1 Introduction

*Idesia polycarpa* Maxim. also known as oil grape, is a deciduous broad-leaved tree belonging to the Flacourtiaceae family (Li et al., 2024a; 2024b). Its tree body is tall, the crown, and the stem shape is straight, and the branches are flourishing, Leaves alternate, thinly leathery or thick papery, ovate or broadly cordate (Cai, 2015). Its wood hardness is moderate, the texture is light and soft, the wood is stable, easy to dry, easy to process, and can be used in construction, furniture and other building materials (Xiong and Liu, 2021). In addition, its pulp oil content is as high as 44.08%, seed oil content of 26.54%, is one of the important woody oilseed species in China, known as “Aerial oil tree” (Fang et al., 2020; Yang et al., 2025). As an emerging woody oil species, *I. polycarpa* contains a variety of fatty acids, linoleic acid content, and the content exceeds 80% (Wang et al., 2011). Related studies have also found that it has antioxidant capacity (Tian et al., 2020) and drug application value (Wang HM, et al., 2023; Wu et al., 2023). In recent years, some researchers have focused on the breeding, cultivation, harvesting, processing and utilization of *I. polycarpa*. There is a lack of studies investigating the effects of specific fertilizer elements on their growth and development.

Nitrogen (N) is an essential nutrient for plant growth and a major limiting factor in many terrestrial ecosystems (Xu and Takah, 2020). For the high-yielding oil tree species *I. polycarpa*, substantial N is required to support its vigorous vegetative and reproductive growth. Identifying the key nutrient constraints on its productivity is therefore crucial to achieving high and stable yields. Similar findings have been reported in the related woody oil crop *Camellia oleifera*, where N is recognized as a critical factor influencing both growth and yield formation (Yuan et al., 2019). Nitrogen deficiency can impair N uptake and assimilation, reduce photosynthetic efficiency, and lead to stunted plant development (Miao et al., 2023). While appropriate N supply enhances leaf photosynthesis, dry matter accumulation, and yield (Duan et al., 2019), excessive application may cause toxicity and inhibit plant growth (Han et al., 2024). Fertilization represents a key management practice for improving soil nutrient status in *I. polycarpa* plantations, and rational N application can significantly promote tree growth and productivity. Thus, evaluating the effects of different N fertilizer rates on the growth and development of *I. polycarpa* is of considerable practical and scientific importance.

The rhizosphere is a microecological environment formed by the interaction between plant roots and soil microorganisms, which plays an important role in plant growth and development (Chen et al., 2025; Jiang RH, et al., 2023). Rhizosphere microorganisms can directly participate in and influence plant physiological processes and can be used as one of the indicators of soil fertility and nutrient abundance (Zhou et al., 2023). The metabolic activity and biological functional diversity index of the rhizosphere microbial community are two important bases for directly characterizing the changes in the activity of the microbial community and explaining the characteristics and status of the microbial ecosystem (Hao et al., 2022). In nature, scientific fertilization can also effectively regulate the cycling, absorption and reuse of carbon sources by soil microorganisms, and significantly increase the growth, metabolic activities and functional diversity of soil microbial communities (Wang SS, et al., 2024). Obviously, soil nutrient status and physical and chemical properties are related to soil microbial diversity.

In addition, soil microbial communities are profoundly influenced by N fertilization (Yao and Li, 2025). N inputs can alter the rhizosphere microbiome, which in turn reshapes the structure and diversity of the surrounding soil microbial community (Zhang et al., 2025; Yin et al., 2024). Studies have shown that N application enhances the carbon utilization capacity of soil microorganisms, thereby increasing microbial richness and functional diversity (Wang XY, et al., 2024). Moreover, an appropriate N application rate can stimulate the abundance of key functional groups such as fungi, actinomycetes, and nitrogen-fixing bacteria, while also regulating soil N transformation processes (Juan et al., 2023).

N is an essential nutrient for the growth and development of *I. polycarpa*. To investigate its effects, a 150-day pot experiment was conducted with four N application levels. The study aimed to examine how N fertilization influences root system growth and rhizosphere soil properties, and how these changes are reflected in the structure, diversity, and functional potential of the associated microbial community. Furthermore, we analyzed the correlations between rhizosphere physicochemical parameters and microbial community composition. The objectives of this study were to determine the N requirements of *I. polycarpa*, optimize its nutrient management, support its cultivation and industrial development, and provide a scientific basis for fertilization and high-yield practices.

## 2 Materials and methods

### 2.1 Overview of the test site

The experimental site is located at the Forestry Experiment Station of Henan Agricultural University, Zhengzhou, Henan Province (113° 42′ E, 34° 43′ N), which belongs to the warm temperate continental monsoon climate, with an average annual temperature of 14.2 °C, an extreme high temperature of 43 °C, an extreme low temperature of - 17.9 °C, an annual precipitation of 650.1 mm, an annual sunshine length of 2400 hours, and a frost-free period of 215 days (Geng et al., 2025).

### 2.2 Test material and fertilizer application

One-year-old *I. polycarpa* were selected as experimental materials. The cultivation soil substrate was a mixture of V (peat soil): V (vermiculite) = 1:1, with an organic matter content of 7.8 mg/kg, a pH of 7.8, an alkali-hydrolysable N content is 25 mg/kg, an available phosphorus content of 48 mg/kg, and an available potassium content of 42 mg/kg. The fertilizers used were urea (N, 46%), potassium superphosphate (P, 12%), and potassium chloride (K, 52%). To ensure that N was the sole limiting factor, phosphorus (P) and potassium (K) were uniformly supplied as a basal fertilizer to satisfy the basic nutritional demands of Chinese quince in all treatments. Thus, the monthly rates were fixed at 1.83 g of P_2_O_5_ and 1.29 g of K_2_O per plant, based on established nutrient requirements and preliminary trials (Wang SS, et al., 2024).

Four N fertilizer treatments were applied: 0 (control), 0.5, 1.0, and 1.5 the recommended amount. Using a randomized block design, each treatment was replicated three times and contained 20 plants per replicate. The specific N application rates for each treatment are shown in **Table 1**. In our pot experiment, the recommended (Rana, 2024) monthly N application rate was 2.4 g per plant. Based on a standard field density of 1,667 plants ha^−1^ (2m × 3m spacing), this corresponds to 4.0 kg N ha^−1^ month^−1^, yielding a total of approximately 24 kg N ha^−1^ over a typical 6-month vigorous growth period.

**Table 1:** Design table of gradient fertilization.

Numbers	Fertilization treatments	Fertilizer (g/strains * times)		
		N(g/plant)	P_2_O_5_(g/plant)	K_2_O(g/plant)
0	N_0_P_2_K_2_	0	1.83	1.29
1	N_1_P_2_K_2_	1.2	1.83	1.29
2	N_2_P_2_K_2_	2.4	1.83	1.29
3	N_3_P_2_K_2_	3.6	1.83	1.29

No nitrogen fertilizer: 0 g (control, CK). 0.5 times the recommended amount: 1.2 g (low nitrogen, LN) Recommended fertilizer amount: 2.4 g. (medium nitrogen, MN), 1.5 times the recommended amount: 3.6 g (high nitrogen, HN).

To match the sustained N demand of *I. polycarpa* throughout its growth season, a monthly split-application strategy was employed. This approach replenishes nutrients in phase with plant uptake, thus reducing leaching risks and maintaining rhizosphere N availability. On May 15, 2023, seedlings were transplanted individually into 29 cm × 25 cm plastic pots. From the start date, fertilizer was applied every 30 days. Plant samples were collected at 30, 60, 90, and 120 days after transplantation. Daily irrigation was provided for 30 minutes via an automatic sprinkler system, with watering split into two 15-minute sessions (morning and evening) during high-temperature periods. Key procedures, including fertilization, indicator measurement, and sampling, were scheduled for the 15th of each month. Root scanner analysis in May confirmed no significant pre-existing variation in root morphology or growth indicators among seedlings, thereby ruling out individual variability prior to treatments.

### 2.3 Soil and plant sample collection

Plant and soil samples were collected monthly (May–September 2023) from each N treatment. Three uniformly grown seedlings per treatment were randomly sampled. Roots were excavated, rinsed, and bagged for scanning; rhizosphere soil was collected. Soil samples were homogenized and split for either freezing (-80 °C) for microbial analysis or air-drying and sieving (2 mm, 0.15 mm) for physicochemical analysis. Each treatment was replicated three times.

### 2.4 Root and soil analysis

#### 2.4.1 Root system index measurement

Fine root morphology was assessed monthly from May to September. Fresh root samples were carefully washed and scanned using a flatbed root scanner (Epson Expression 12000XL, Japan) at a resolution of 600 dpi to obtain high-quality digital images. The resulting images were then analyzed with the WINRHIZO TRON root analysis system (Horde Technologies, China) to quantify key morphological parameters, including total root length, average root diameter, root volume, and root surface area.

#### 2.4.2 Measurement of physical and chemical properties of rhizosphere soil

Soil pH was assessed in a soil-water slurry (1:2.5 w/v) using 10 g of air-dried, 2-mm sieved soil and CO_2_-free distilled water, following a 30-min equilibration period. Measurements were taken with a pH meter (PHS-3E, Shanghai Yidian Scientific Instrument Co., Ltd., China).

The following soil properties were determined: available nitrogen (AN) by the alkaline hydrolysis diffusion method; available phosphorus (AP) by sodium bicarbonate extraction followed by molybdenum-antimony anti-colorimetric analysis using a UV spectrophotometer (E1, Peakch Instruments Co., Ltd., China); and available potassium (AK) by ammonium acetate extraction and flame photometry (WGH6400, Shanghai Lichen Bangxi Instrument Technology Co., Ltd., China). Soil organic matter (SOM) and total carbon (TC) were quantified using the potassium dichromate oxidation-external heating method (Feng et al., 2022; Sun, 2011; Kiremit et al., 2024).

#### 2.4.3 DNA extraction and high-throughput sequencing of rhizosphere soil

Total DNA was extracted by modified Cetyltrimethylammonium Bromide (CTAB) method (Zhou et al., 2021) and detected by 1% agarose gel electrophoresis. 16Sr RNA and ITS of total DNA were amplified by Polymerase Chain Reaction (PCR) using barcoded specific primers. The PCR amplification was carried out in a 50 μL PCR amplification system. The amplification reaction conditions were pre-denaturation at 98 °C for 5min, denaturation at 98 °C for 30s, annealing at 50 °C for 30s, extension at 72 °C for 30s, 25 cycles, and extension at 72 °C for 5min. PCR products were detected by agar gel electrophoresis with volume fraction of 2%, and the remaining samples were stored at 4 °C for later use. Bacterial forward primers: 5′-AACMGGATTAGATAC-CCKG-3′. Reverse primers: 5′-ACGTCATCCCCACCACT-TCC-3′. Fungal forward primers: 5′-CTTGGTCATTTAGAG-GAAGTAA-3′. Reverse primers: 5′-GCTGCGTTCT-TCATCGATGC-3′ (Du et al., 2024, Jiang X, et al., 2023). There were 3 replicates in each group, and the corresponding amplified regions were 16S V5 and V7 primers, which could help identify bacterial diversity, ITS1 and ITS4 primers can help identify fungal diversity. The amplified products were Paired-end sequenced on the community DNA fragments on the Illumina platform, and the sequencing process was completed by Shanghai Parsonage Biotech (Shanghai, China).

### 2.5 Data analysis

Prior to parametric tests, the normality of all data—including plant root morphological traits (length, diameter, volume, and surface area) and soil physicochemical properties—was assessed using the Shapiro–Wilk test. Data were preprocessed in Microsoft Excel 2021 (https://www.microsoft.com) and analyzed with IBM SPSS Statistics v.26 (https://www.ibm.com). Normally distributed data were compared among treatments using one-way analysis of variance (ANOVA). When ANOVA indicated significant differences (*P* < 0.05), the Waller–Duncan post-hoc test was applied to determine differences between specific groups.

The primer fragments of the sequence were removed using QIIME 2 2019.4 version (https://docs.qiime2.org/2019.4/tutorials/), and the sequences without matching primers were discarded (Martin, 2011). The DADA2 plug-in was used to perform quality control, denoising, splicing, and mosaic removal on the sequences (Callahan et al., 2016). The OTUs feature sequences and OTU tables were merged, and the data of the composition distribution of each sample at each classification level was visualized, and the clustering results were presented at a similarity level of 97% (Barnes et al., 2020). The species alignment and annotation were performed using the RDP classifier (version 2.11) software, and the annotation results with a confidence interval greater than 0.8 were retained. The Chao 1 index was used to reflect species richness, the Shannon and Simpson indices were used to reflect the diversity of microbial community. The Chao1 (Chao, 1984), Simpson (Simpson, 1949), and Shannon (Shannon, 2001) abundance indexes were calculated using scikit-bio, and the community structure was statistically analyzed at each classification level to obtain the microbial community structure composition. To identify the key environmental drivers of soil bacterial and fungal community shifts under varying N fertilization regimes, redundancy analysis (RDA) was performed. This analysis assessed the relationships between microbial community structures (for both bacteria and fungi) and measured soil physicochemical properties, including pH, AN, and AP (Li et al., 2020). The KEGG database and MetaCyc database were used to predict bacterial and fungal functions. The bacterial community was analyzed using the KEGG database, and the results were categorized into six primary metabolic pathways, including Cellular Processes, Environmental Information Processing, Genetic Information Processing, Human Diseases, Metabolism and organismal systems. By comparing the fungal community with the MetaCyc database, we annotated a total of five primary metabolic pathways and 29 secondary metabolic pathways (Douglas et al., 2020).

## 3 Results

### 3.1 Effects of different N levels on *I. polycarpa* root density

**Figure 1:**
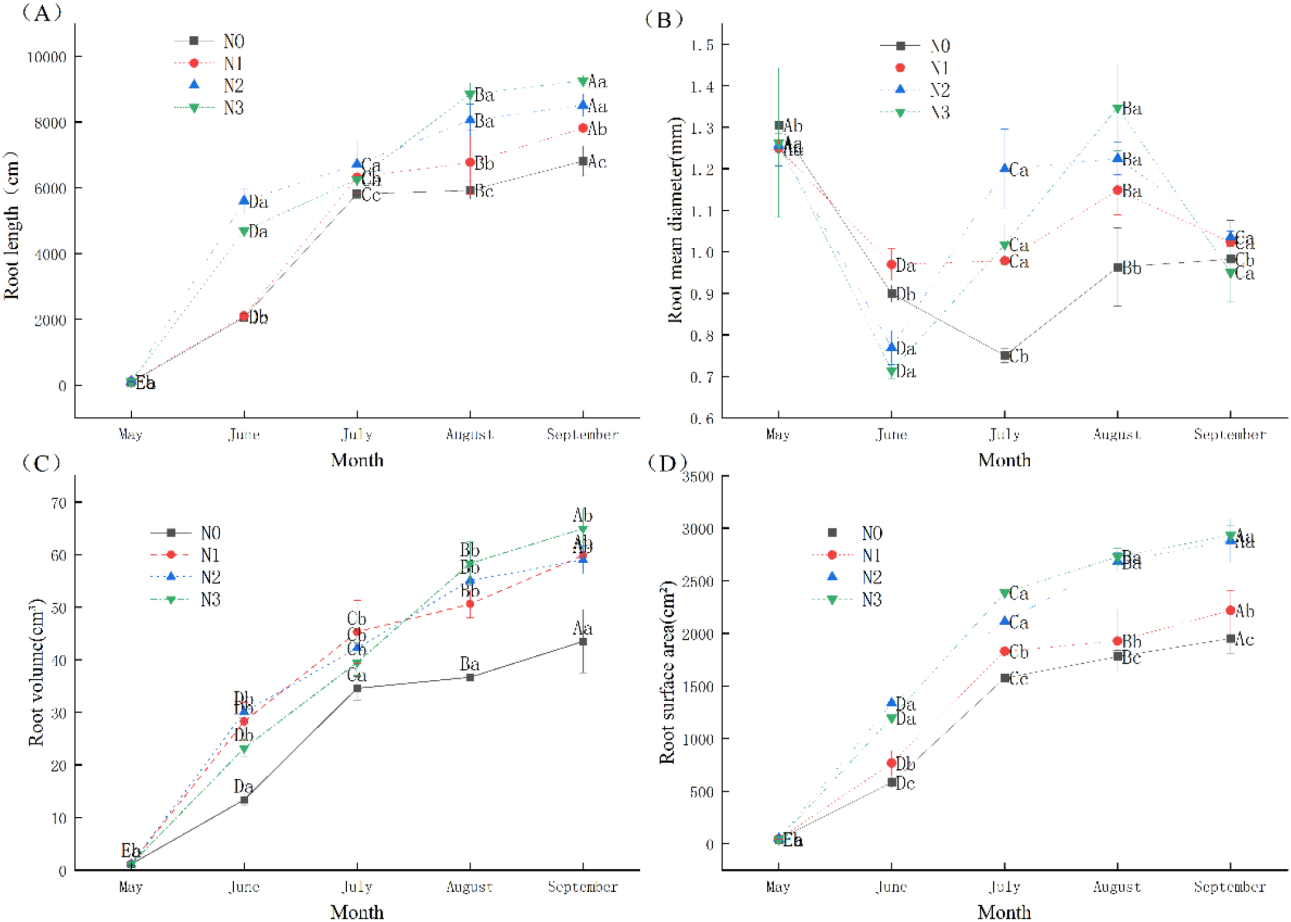
Effects of different concentrations of N fertilizer on root development of *I*. *polycarpa*. **(A)** The effects of different N concentrations on root length of *I*. *polycarpa*. **(B)** The effects of different N concentrations on root mean diameter of *I*. *polycarpa*. **(C)** The effects of different N concentrations on root volume of *I*. *polycarpa*. **(D)** The effects of different N concentrations on root surface area of *I*. *polycarpa*. Different uppercase letters after the same column data indicate significant (*p < 0.05*) differences between different stress times for the same treatment, and different lowercase letters after the same column data indicate significant (*p <0.05*) differences between treatments for the same period.

The application of N fertilizer significantly influenced the growth and development of root system of *I. polycarpa*. There were significant differences (*p* < 0.05) in root length, root average diameter, root volume and root surface area among fertilization treatments (**Figure 1**). The root morphology of *I. polycarpa* exhibited consistent dynamic changes throughout the growing season (May to September). Root length, volume, and surface area increased significantly over time, with root length growing from less than 120 cm in May to over 6800 cm in September. The pattern for mean root diameter differed: it remained relatively high in May (approx 1.26-1.31 mm), then decreased significantly to its lowest values in June–July (0.71-0.98 mm), before gradually recovering in August–September. Regarding fertilization effects, the MN and HN treatments significantly outperformed the CK in terms of root length and surface area in most measurement months, particularly after June, indicating that nitrogen application effectively enhanced root morphogenesis. The In review results of multiple comparisons showed that the root length, root mean diameter, root volume and root surface area in different months were significantly different among different treatment groups (*p* < 0.05).

### 3.2 Effects of different N levels on soil physical and chemical properties

**Figure 2:**
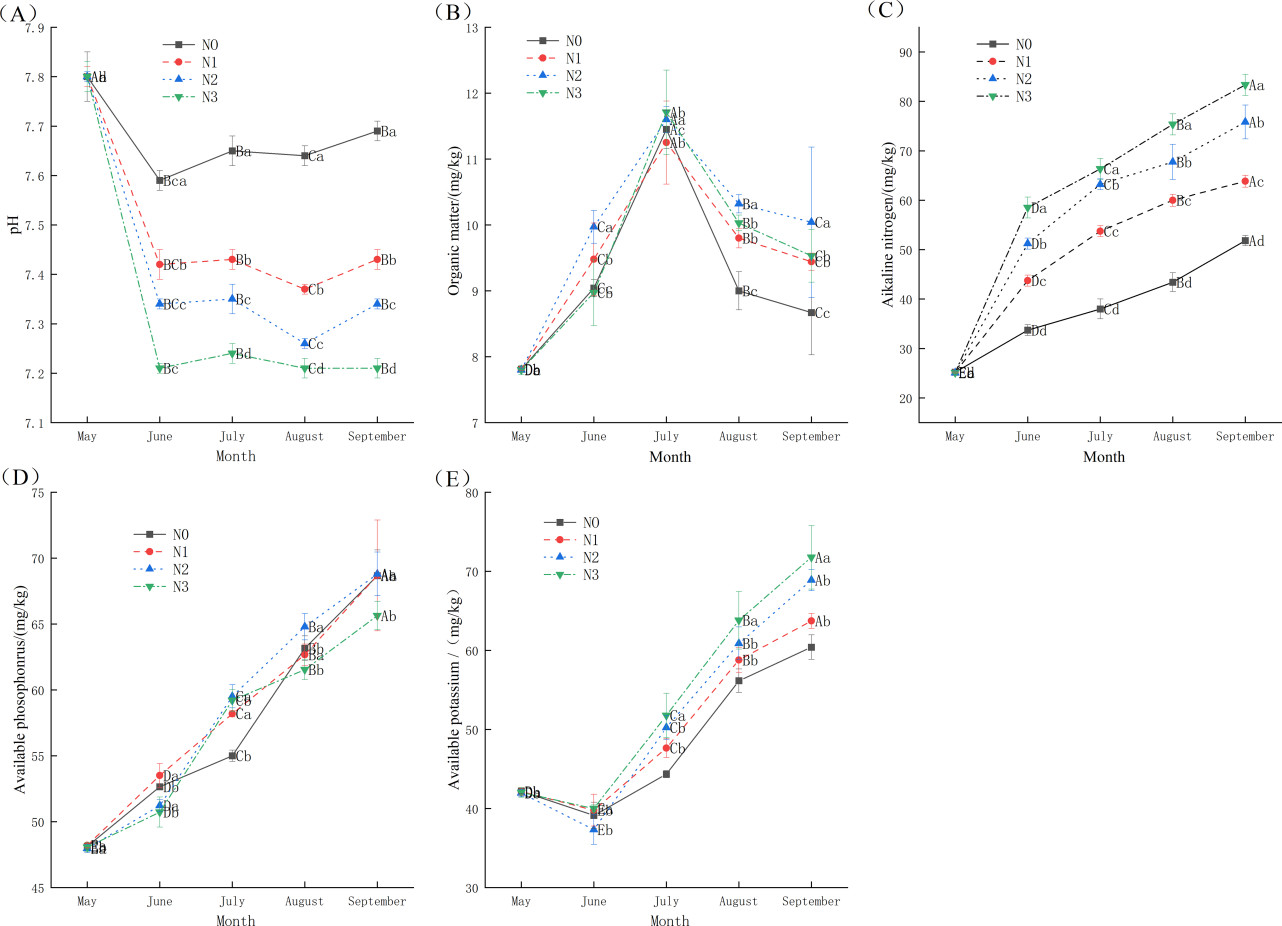
The effect of different concentrations of N fertilizer on the physical and chemical properties of soil for *I*. *polycarpa*. **(A)** The effects of different N concentrations on soil pH of *I*. *polycarpa*. **(B)** The effects of different N concentrations on soil SOM of *I*. *polycarpa*. **(C)** The effects of different N concentrations on soil AN of *I*. *polycarpa*. **(D)** The effects of different N concentrations on soil AP of *I*. *polycarpa*. **(E)** The effects of different N concentrations on soil AK of *I*. *polycarpa*. Different uppercase letters after the same column data indicate significant (*p <0.05*) differences between different stress times for the same treatment, and different lowercase letters after the same column data indicate significant (*p < 0.05*) differences between treatments for the same period.

The application of N fertilizer significantly influenced physical and chemical properties of root soil of *I. polycarpa* (**Figure 2**). There were significant differences (*p* < 0.05) in SOM, AN, AP and AK among fertilization treatments. Throughout the growing season (May to September), the contents of soil AN, AP, and AK generally increased, peaking in September. In contrast, soil organic matter content first increased and then decreased, reaching its maximum in July. Compared to the CK, MN and HN treatments consistently resulted in significantly higher levels of AN, SOM, and AP, with differences being most pronounced during the mid-to-late growing period. These findings suggest that moderate nitrogen addition not only promotes plant growth but also enhances rhizosphere soil fertility. Soil pH remained stable across all treatments.

### 3.3 Microbial community structure in rhizosphere soil of *I. polycarpa*

#### 3.3.1 Soil bacterial community structure

**Figure 3:**
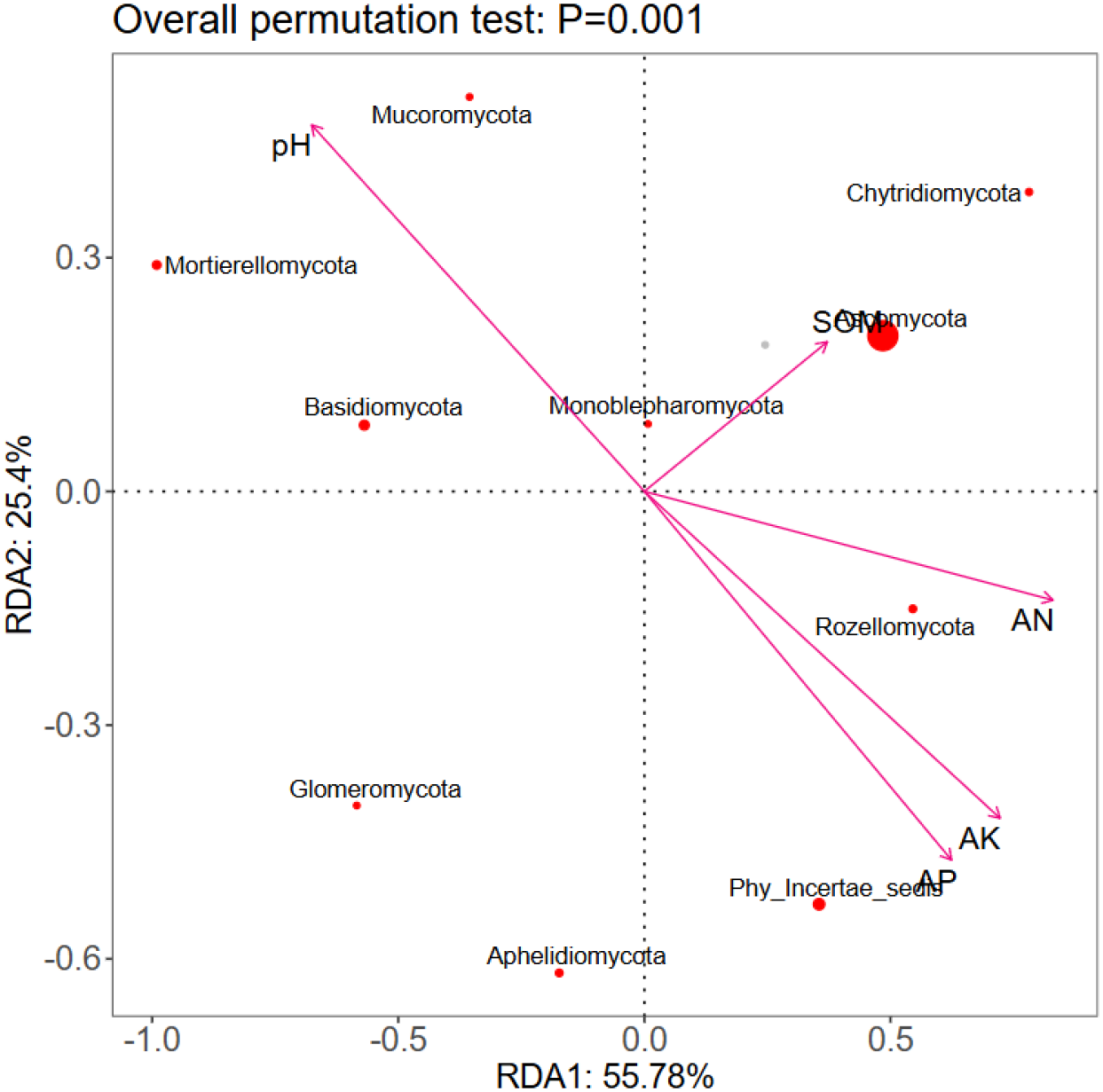
RDA diagram of soil environmental factors and fungi communities.

At the phylum level, the bacterial community in the rhizosphere soil of *I. polycarpa* was primarily dominated by Proteobacteria (32.73%-41.05%), Chloroflexota (8.46% ~ 16.88%), Acidobacteria (10.00% ~ 20.99%), Gemmatimonadetes (6.84% ~ 13.64%), Actinobacteria (6.39% ~ 13.50%), Bacteroidetes (3.78% ~ 5.96%), Myxococcota (1.76% ~ 4.38%), Patescibacteria (1.35% ~ 2.96%), Verrucomicrobioa (0.67% ~ 2.51%), Planctomycetoa (0.53% ~ 2.65%). A histogram of the top twenty genera with relative abundance of rhizosphere bacteria was made (**Figure 3**), and the genera with an average relative abundance of more than 1% were *A4b* (4.05%), *Vicinamibacteraceae* (4.02%), *Subgroup _ 10* (3.17%), *Gemmatimonas* (1.94%), *Hirschia* (1.59%), *SWB02* (1.55%), *SBR1031* (1.50%), *KD4-96* (1.30%), *JG30-KF-CM45* (1.23%), *R7C24* (1.14%), *Iamia* (1.13%), *TRA3-20* (1.08%) and *IMCC26256* (1.08%).

#### 3.3.2 Soil fungal community structure

At the phylum level, the rhizosphere fungal community of *I. polycarpa* was predominantly composed of Ascomycota (81.18% ~ 93.25%), Basidiomycota (0.41% ~ 3.87%) and Mortierellomycota (0.01% ~ 3.56%). The genera with an average relative abundance of more than 1% were *Humicola* (66.67%), *Pseudogymnoascus* (7.94%), *Penicillium* (2.17%), *Botryotrichum* (2.13%) and *Mycothermus* (1.52%).

#### 3.3.3 Diversity of soil bacteria

The bacterial alpha(*α*) diversity in the rhizosphere soil was analyzed across different N application levels during the months of June, July, August, and September. Statistical analysis revealed that N fertilization had a significant effect on these diversity indices at specific growth stages (**Table 2**). Soil bacterial abundance and diversity under the CK treatment declined continuously throughout the growth period. In contrast, those under the LN and MN treatments showed an overall trend of an initial increase followed by a decrease, peaking in July, and HN level decreased first and then increased and then decreased, reaching the maximum value in August. The α-diversity index of soil bacteria in June showed a gradual increase with rising nitrogen fertilizer application rates, peaking at the HN level. The Chao1 index exhibited the order HN > MN > LN, representing increases of -1.82%, -3.87%, and -8.97% compared to the CK. The Shannon index showed LN > HN > MN, with increases of 1.49%, 1.1%, and -0.6% compared to CK. The Simpson index exhibited LN > HN > MN, with increases of 0.02%, -0.01%, and -0.58% relative to CK. In July, soil bacterial α-diversity indices showed an initial increase followed by a decline. The Chao1, Shannon, and Simpson indices exhibited the order LN > MN > HN, with increases of 19.59%, 18.87%, and -9.03%, compared to CK; 1.05%, 0.19%, and -2.95%; 0.01%, 0.01%, and -0.07%, respectively. This indicates that a small amount of nitrogen fertilizer can more effectively promote soil bacterial diversity during this stage. In August, soil bacterial α-diversity indices gradually increased with rising nitrogen fertilizer application rates, peaking at the HN level. The Chao1, Shannon, and Simpson indices followed the order HN > MN > LN, showing increases of 7.85%, 7.62%, and -1.45%; 3.46%, 1.38%, and 0.89%; 0.09%, 0.06%, 0.05%, respectively. In September, the soil bacterial α-diversity index first increased then decreased with rising nitrogen fertilizer application rates, reaching its maximum at the LN level. The Chao1 and Shannon indices showed LN > MN > MN, increasing by 14.82%, -10.71%, -13.36%; 0.49%, -0.13%, -0.29%, respectively. The Simpson index showed HN > LN > MN, with increases of 0.01%, 0%, and -0.04% compared to CK. This indicates that low nitrogen fertilizer application readily promotes soil bacterial richness and diversity during this stage.

**Table 2:** The *α* diversity indices of soil bacteria.

Month	Treatment	Chao1 index	Shannon index	Simpson index
June	N0	2911.96 ± 163.19Ba	10.05 ± 0.03Cb	0.9976 ± 0.0001Ca
	N1	2650.67 ± 64.80Ba	10.20 ± 0.03Ca	0.9978 ± 0.0001Ca
	N2	2799.14 ± 132.82Ba	9.99 ± 0.03Cb	0.9918 ± 0.0003Cb
	N3	2858.88 ± 90.84Ba	10.16 ± 0.02Cb	0.9975 ± 0.0001Ca
July	N0	2881.22 ± 104.44Aa	10.52 ± 0.04Ab	0.9987 ± 0.0001Aa
	N1	3445.67 ± 166.86Aa	10.63 ± 0.02Aa	0.9988 ± 0.0001Aa
	N2	3424.99 ± 72.87Aa	10.54 ± 0.04Ab	0.9988 ± 0.0001Ab
	N3	2730.99 ± 219.48Aa	10.21 ± 0.07Ab	0.9980 ± 0.0001Aa
August	N0	2751.82 ± 144.10Ba	10.11 ± 0.04Bb	0.9978 ± 0.0001Ba
	N1	2711.83 ± 192.76Ba	10.20 ± 0.03Ba	0.9983 ± 0.0001Ba
	N2	2961.44 ± 275.48Ba	10.25 ± 0.02Bb	0.9984 ± 0.0001Bb
	N3	2967.80 ± 165.92Ba	10.46 ± 0.03Bb	0.9987 ± 0.0001Ba
September	N0	2736.85 ± 24.31Ba	10.28 ± 0.07Bb	0.9985 ± 0.0001ABa
	N1	3142.51 ± 225.11Ba	10.33 ± 0.04Ba	0.9985 ± 0.0001ABa
	N2	2371.13 ± 79.36Ba	10.15 ± 0.05Bb	0.9981 ± 0.0001ABb
	N3	2443.86 ± 74.23Ba	10.25 ± 0.02Bb	0.9986 ± 0.0002ABa

Different uppercase letters indicate significant differences (*p* < 0.05) among sampling times within the same treatment; different lowercase letters indicate significant differences (*p* < 0.05) among treatments at the same sampling time.

#### 3.3.4 Diversity of soil fungi

The composition of soil fungal communities in *I. polycarpa* rhizosphere was significantly influenced by nitrogen application rates (**Table 3**). Except for the richness and diversity at CK level increased first and then decreased with growth, the diversity and richness at LN, MN and HN levels decreased as a whole, reaching the maximum value in July. The α-diversity indices of soil fungi in June showed an initial increase followed by a decrease with rising nitrogen fertilizer application rates, peaking at the MN level. Compared to the CK, the Chao1, Shannon, and Simpson indices increased by 21.64%, 46.1%, and 23.42%, respectively; 11.93%, 44.92%, and -4.56%; 9.58%, 32.35%, and -11.6%, respectively. In July, the Chao1 index in soil showed MH > LN > HN, with increases of -22.14%, -22.70%, and -24.83% compared to CK. Shannon and Simpson indices showed LN > MN > HN, with increases of -4.50%, -17.46%, -37.84%; -0.67%, -7.84%, -33.81% compared to CK. This indicates that soil fungal diversity decreased with increasing nitrogen fertilizer application in July. In August, the α-diversity indices of soil fungi exhibited a “double-peak” pattern of initial decline, subsequent increase, and final decrease. The Chao1, Shannon, and Simpson indices showed MN > LN > HN, with increases of 14.78%, 11.26%, -9.79%; -4.69%, -12.61%, -19.94%; 0.15%, -11.84%, -17.93% compared to CK respectively. indicating that both insufficient and excessive nitrogen fertilizer suppressed soil fungal diversity during this period; In September, the Chao1 index for soil fungi showed LN > MN > HN, increasing by 18.83%, 12.44%, and 10.14% compared to CK. The Shannon and Simpson indices exhibited MN > HN > LN, increasing by 25.26%, 1.02%, -0.14%; 28.34%, 1.74%, -4% compared to CK respectively.

**Table 3:** The *α* diversity indices of soil fungi.

Month	Treatment	Chao1 index	Shannon index	Simpson index
June	N0	198.24 ± 6.37Ab	2.85 ± 0.03Ab	0.5792 ± 0.0077Ab
	N1	241.13 ± 1.45Aa	3.19 ± 0.01Ac	0.6347 ± 0.0019Ac
	N2	289.55 ± 4.52Ab	4.13 ± 0.05Aa	0.7666 ± 0.0071Aa
	N3	244.66 ± 2.47Ab	2.72 ± 0.03Ad	0.512 ± 0.0049Ad
July	N0	250.10 ± 3.58Bb	3.78 ± 0.06Ab	0.7045 ± 0.0098Ab
	N1	193.33 ± 13.61Bb	3.61 ± 0.03Ac	0.6998 ± 0.0077Ac
	N2	194.74 ± 6.24Bb	3.12 ± 0.01Aa	0.6493 ± 0.0019Aa
	N3	188.10 ± 5.01Bb	2.35 ± 0.01Ad	0.4663 ± 0.0078Ad
August	N0	175.41 ± 3.97Cb	3.41 ± 0.10Bb	0.6613 ± 0.0213Bb
	N1	158.23 ± 2.83Ca	2.73 ± 0.05Bc	0.5427 ± 0.0054Bc
	N2	201.34 ± 2.13Cb	3.25 ± 0.05Ba	0.6223 ± 0.0066Ba
	N3	195.16 ± 8.41Cb	2.98 ± 0.06Bd	0.583 ± .0108Bd
September	N0	156.33 ± 11.22Db	2.93 ± 0.09Bb	0.5682 ± 0.0156Bb
	N1	185.76 ± 4.91Da	2.89 ± 0.12Bc	0.5455 ± 0.0181Bc
	N2	175.78 ± 8.38Db	3.67 ± 0.04Ba	0.7292 ± 0.0059Ba
	N3	172.18 ± 6.77Db	2.90 ± 0.09Bd	0.5781 ± 0.0137Bd

Different uppercase letters indicate significant differences (*p* < 0.05) among sampling times within the same treatment; different lowercase letters indicate significant differences (*p* < 0.05) among treatments at the same sampling time.

#### 3.3.5 Redundancy analysis of soil environmental factors and bacterial communities

**Figure 4:**
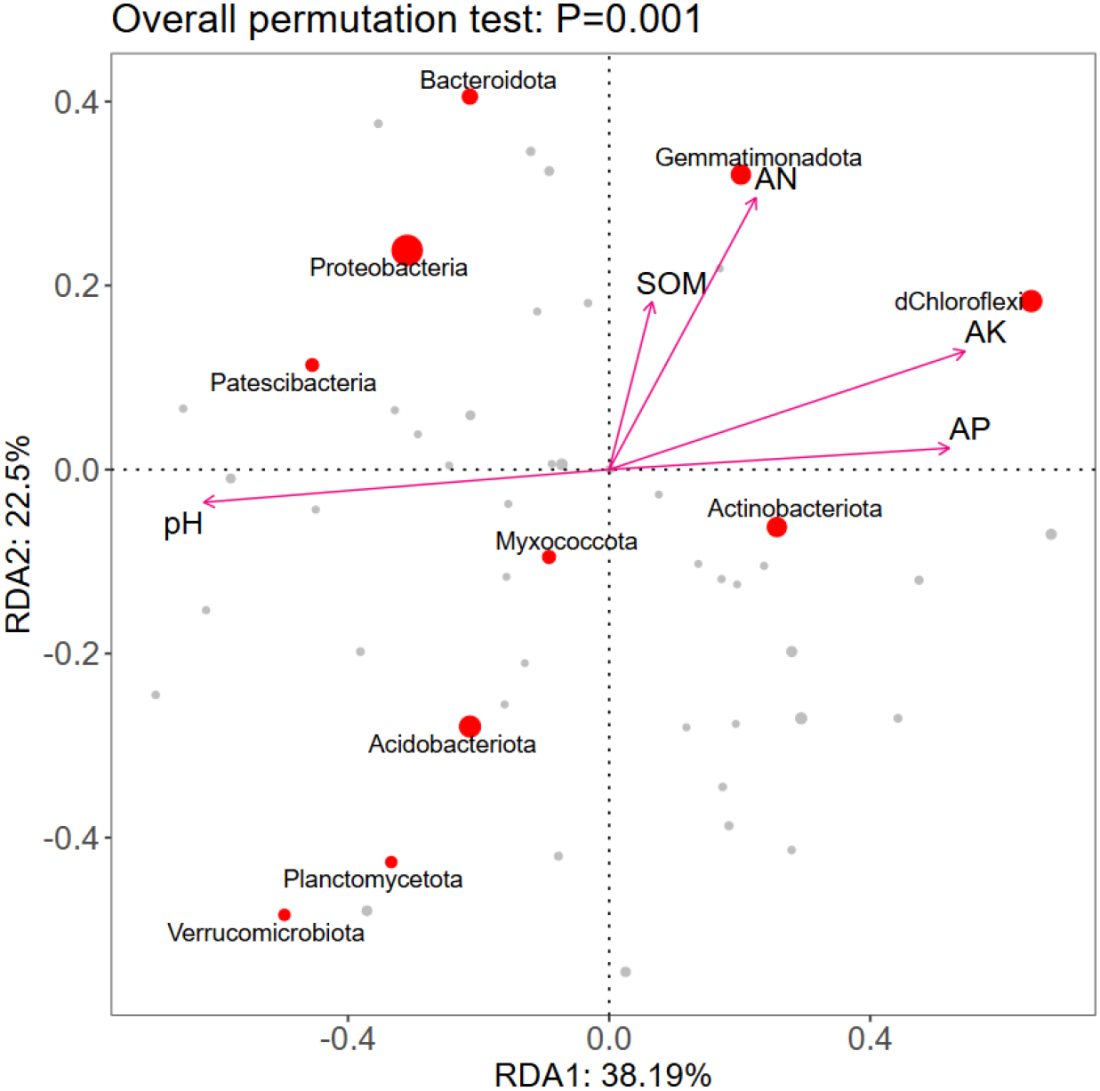
RDA diagram of soil environmental factors and bacterial communities.

The first and second axes account for 60.69% of the variables, and the two axes may reflect the influence of soil factors on the soil bacterial communities. The RDA results showed (**Figure 4**) that AN (*r* = 0.4937, *p* = 0.0005), AP (*r* = 0.6935, *p* = 0.0005), AK (*r* = 0.7448, *p* = 0.0005), pH (*r* = 0.8270, *p* = 0.0005) were the key factors leading to differences in soil bacterial communities.

#### 3.3.6 Redundancy analysis of soil environmental factors and fungi communities

The first and second axes accounted for 81.04% of the variables, and the two axes may reflect the influence of soil factors on soil fungal communities. RDA results showed (**Figure 3**) that AN (*r* = 0.6144, p = 0.0005), AP (*r* = 0.5714, *p* = 0.0005), AK (*r* = 0.6097, *p* = 0.0005), pH (*r* = 0.6009, *p* = 0.0005) and SOM (*r* = 0.3053, *p* = 0.0005) were the key factors leading to differences in soil fungi commun.

#### 3.3.7 Predictive analysis of soil microbial community function

**Figure 5:**
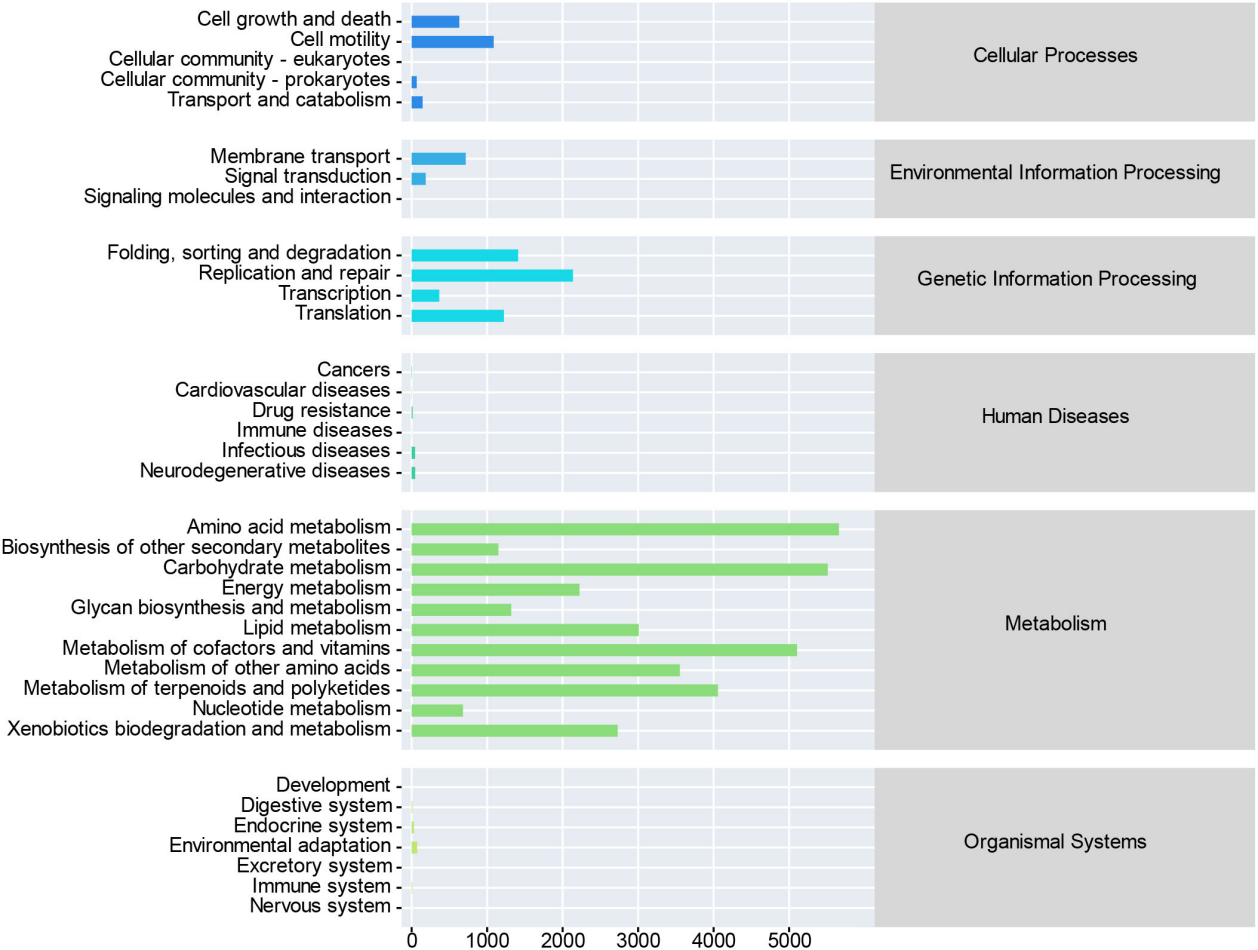
Prediction of soil bacterial function.

Functional prediction based on the KEGG database indicated that metabolism was the predominant primary metabolic pathway in the soil bacterial community across all N levels and sampling months (**Figure 5**). The relative abundance of metabolic functions remained consistently high, ranging from 80.05% to 81.59% throughout the experimental period (June to September), with no pronounced variation observed among different N treatments.

**Figure 6:**
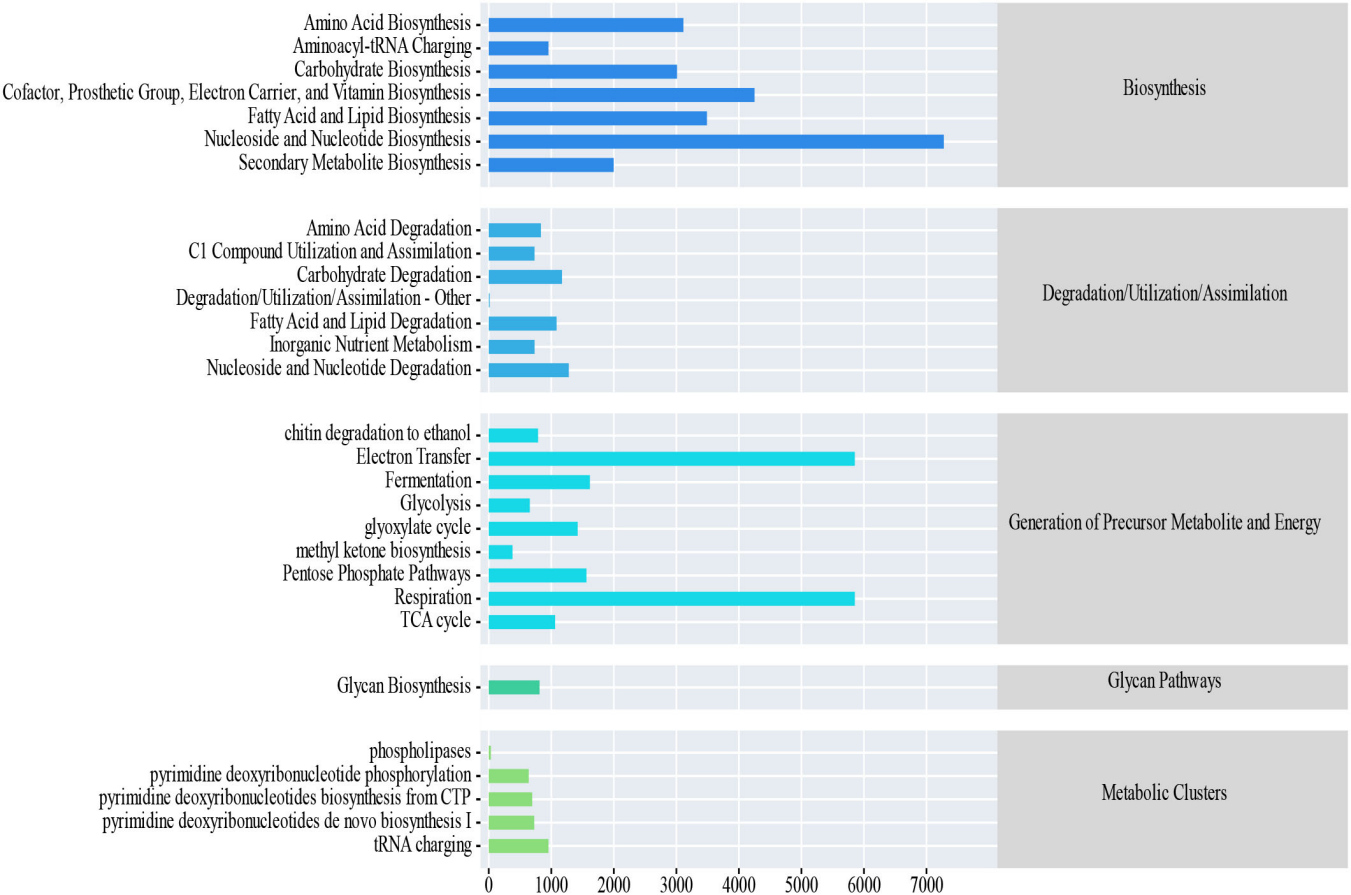
Prediction of soil fungi function.

Functional prediction of the fungal community via the MetaCyc database revealed that the predominant functional categories were “Biosynthesis” and “Generation of Precursor Metabolite and Energy” (**Figure 6**). Across all N levels and sampling months (June to September), the relative abundance of biosynthetic pathways remained consistently high (approximately 44.8%–47.0%), while that of precursor metabolite and energy generation pathways ranged from approximately 31.2% to 37.0%. No pronounced temporal trend or significant differences among N treatments were observed for either functional category.

## 4 Discussion

### 4.1 Effects of different N levels on root indexes of *I. polycarpa*

The growth-promoting effect of N (up to the optimum level) increases cytokinin production, which subsequently affects cell wall elasticity (Bloom et al., 2006). The results demonstrated that the HN treatment exerted the most pronounced positive effects on root morphological parameters, including root volume, surface area, and length. With the exception of average root diameter, all measured root indices across fertilization treatments were superior to those of the CK group, indicating that nitrogen application significantly promoted the growth of *I. polycarpa* seedlings. This finding aligns with the conclusion reported by Duan et al. (2017) in their study on *Ormosia henryi* Prain. Overall, the growth performance across treatments followed the order HN > MN > LN > CK, suggesting a general positive correlation between seedling growth status and nitrogen application rate within the tested range. However, it is noteworthy that excessively high fertilization rates can potentially induce toxic effects, inhibiting seedling growth or even causing seedling burn, as evidenced in other studies (e.g., Yang et al., 2024). The optimal fertilizer ratio may thus vary with plant species and soil conditions, a point supported by the work of Li et al (Li et al., 2022). Regarding growth dynamics, this study found that May to July represents the rapid growth phase for *I. polycarpa* seedlings, during which root morphological indicators significantly improved. This period coincides with the most favorable growth conditions (Jia et al., 2023). The observed growth slowdown from July to September may be attributed to emerging seasonal stresses such as summer heat, a factor that could inhibit root development in *I. polycarpa* (Zeng et al., 2025; Si et al., 2022).

### 4.2 Effects of different N levels on soil microbial community

The soil microbial community represents a critical component of plant microecology, with its composition and function shaped by multiple factors including tree species and anthropogenic disturbance. In this study, the diversity of the soil microbiome associated with *I. polycarpa* varied significantly with sampling time and N application rate. Specifically, bacterial richness in unfertilized soil gradually decreased over the growing season. In contrast, soils receiving LN and medium MN levels exhibited an initial increase in bacterial richness, peaking in July during the period of most rapid plant growth, followed by a decline. Overall, HN levels appeared to suppress bacterial richness. Fungal richness showed an opposite trend: it increased gradually in CK soil but decreased under N fertilization. This suggests that LN and MN application may enhance soil fungal richness in the short term, whereas prolonged or HN fertilization tends to reduce fungal richness and diversity, consistent with findings reported in related studies (Li and Bo, 2024; Anna et al., 2023).

The taxonomic composition of the rhizosphere microbial community varied across different growth stages of *I. polycarpa*, with shifts in the relative abundance of specific bacterial and fungal taxa. At the phylum level, the bacterial community was consistently dominated by *Proteobacteria*, *Chloroflexota*, *Acidobacteria*, and *Actinobacteria*, which collectively accounted for >70% of the total relative abundance (Nayak et al., 2024). These phyla are known for their strong environmental adaptability (Pang et al., 2022). Although the dominant bacterial phyla did not differ significantly over time, their relative abundances varied, likely due to factors such as soil type and plant physiology. This pattern aligns with findings from Tang et al. (2020) in fertilized rice systems, where *Proteobacteria* remained the most abundant phylum. Functionally, these dominant groups play critical roles in soil nutrient cycling: *Proteobacteria* are involved in carbon and nitrogen metabolism (Tan et al., 2019); *Chloroflexota* contribute to polysaccharide degradation and soil organic matter (SOM) accumulation (Zhang et al., 2024); *Acidobacteria* participate in litter decomposition and SOM formation (Chen et al., 2023); and *Actinobacteria* secrete extracellular enzymes for carbon degradation and produce antimicrobial metabolites (Zeng et al., 2023).Notably, N application significantly influenced the relative abundance of specific bacterial taxa at given time points. For instance, in June, the abundances of *Actinobacteria* and the genus *Blastomonas* were significantly higher under high nitrogen treatment compared to other levels. This is consistent with studies reporting that fertilization can increase the abundance of *Actinobacteria* (Li and Bo, 2024) and *Blastomonas* (Wu et al., 2024).

In the soil fungal community of *I. polycarpa*, *Ascomycota* was the dominant phylum, while the relative abundances of other phyla were considerably lower. This finding aligns with previous reports that *Ascomycota* and *Basidiomycota* typically dominate the fungal communities in forest soils (Qiao et al., 2023; Wang TL, et al., 2023). Fungi within *Ascomycota* are primarily saprophytic, playing a crucial role in decomposing plant residues and organic matter, thereby driving internal soil nutrient cycling (Yang et al., 2022). Notably, the relative abundance of the genus *Humicola* differed significantly among fertilization levels. *Humicola* is commonly associated with soils rich in organic matter (Ma et al., 2023). Given that soil organic matter content often correlates positively with nitrogen application rate (Mao et al., 2024), the significantly higher abundance of *Humicola* under HN likely reflects this relationship.

## 5 Conclusions

N fertilization significantly promoted the root morphological development of *I. polycarpa*. In the rhizosphere soil, the contents of AN, AP and AK generally increased with HN application rates. As the growing season progressed, SOM content exhibited an initial increase followed by a decline. In contrast, soil pH decreased consistently throughout the entire growth period.

In this study, the LN treatment and MN treatment promoted soil bacterial α diversity more effectively than the HN treatment, differing from the conventional wisdom that “more nutrients are always better.” Specifically, soil microbial community structure and diversity of *I. polycarpa* were affected by fertilizer application at different N levels. During the rapid growth phase, low to moderate levels of N fertilizer have the best effect on promoting bacterial abundance and diversity. Applying N fertilizer during the slow growth phase reduces bacterial abundance and diversity, Throughout the growing season, N fertilizers reduce the richness and diversity of soil fungi. Therefore, an N application rate of 1.2–2.4 g per plant per month is recommended for optimal growth during the early rapid growth phase of *I. polycarpa*. This study provides new evidence for the application of nitrogen fertilizers in the field of soil microbiology. It clarifies the value of optimizing nitrogen fertilizer application strategies to achieve microbial community health and sustainable soil utilization.

While this pot-based study provides insights under controlled conditions, its conclusions may be subject to interannual climate variability and thus require validation through multi-year field experiments to confirm their broader applicability. Furthermore, although high-throughput sequencing revealed shifts in microbial community composition, it lacked the functional resolution to elucidate underlying gene expression or microbial interaction mechanisms. Future research should focus on (1) establishing long-term field trials to assess the temporal dynamics of soil microbial communities under different nitrogen regimes; (2) applying metagenomic approaches to characterize functional changes in key microbial groups and their drivers; (3) integrating microbial community indicators with soil nutrient cycling rates to develop predictive models linking microbial structure to ecosystem function.

## Data Availability

The original contributions presented in the study are included in the article/supplementary material. Further inquiries can be directed to the corresponding authors.
